# Osteochondral Regeneration Using Adipose Tissue-Derived Mesenchymal Stem Cells

**DOI:** 10.3390/ijms21103589

**Published:** 2020-05-19

**Authors:** Daiki Murata, Ryota Fujimoto, Koichi Nakayama

**Affiliations:** 1 Center for Regenerative Medicine Research, Faculty of Medicine, Saga University, Honjo-machi, Saga 840-8502, Japan; R-FUJIMOTO@nakayama-labs.co.jp (R.F.); nakayama@me.saga-u.ac.jp (K.N.); 2 Department of Oral and Maxillofacial Surgery, Faculty of Medicine, Saga University, Nabeshima 5-1-1, Saga 849-8501, Japan

**Keywords:** adipose tissue-derived mesenchymal stem cells, cell construct, Kenzan method, mold method, osteoarthritis, osteochondral regeneration, scaffold-free, spheroid

## Abstract

Osteoarthritis (OA) is a major joint disease that promotes locomotor deficiency during the middle- to old-age, with the associated disability potentially decreasing quality of life. Recently, surgical strategies to reconstruct both articular cartilage and subchondral bone for OA have been diligently investigated for restoring joint structure and function. Adipose tissue-derived mesenchymal stem cells (AT-MSCs), which maintain pluripotency and self-proliferation ability, have recently received attention as a useful tool to regenerate osteocartilage for OA. In this review, several studies were described related to AT-MSC spheroids, with scaffold and scaffold-free three-dimensional (3D) constructs produced using “mold” or “Kenzan” methods for osteochondral regeneration. First, several examples of articular cartilage regeneration using AT-MSCs were introduced. Second, studies of osteochondral regeneration (not only cartilage but also subchondral bone) using AT-MSCs were described. Third, examples were presented wherein spheroids were produced using AT-MSCs for cartilage regeneration. Fourth, osteochondral regeneration following autologous implantation of AT-MSC scaffold-free 3D constructs, fabricated using the “mold” or “Kenzan” method, was considered. Finally, prospects of osteochondral regeneration by scaffold-free 3D constructs using AT-MSC spheroids were discussed.

## 1. Introduction

Articular cartilage constitutes hyaline cartilage, which is generally encompassed by perichondrium consisting of collagen fibers and early mesenchymal progenitor cells that can differentiate into chondrocytes [1]. However, articular cartilage is instead covered by horizontally arranged collagen-containing proteoglycans, such as lubricin [2]. Therefore, the articular surface exhibits lubricity but does not possess self-healing ability, owing to the absence of vascularity and perichondrium [3]. Cartilage defects are frequently observed in young and active patients, and the repaired tissue is fibrous due to the poor self-healing capacity of articular cartilage [4,5]. Notably, this fibrous tissue does not have the functional properties of natural hyaline cartilage. Thus, the defect leads to cartilage degradation and osteochondral lesions, defined as subchondral bone sclerosis/deformation, which usually develops into osteoarthritis (OA) [6]. OA constitutes a major joint disease that causes movement disorders in the middle- and old-age [7,8,9], and the associated disabilities can reduce the quality of life. Therefore, surgical strategies to rebuild both bone and cartilage to restore joint structure and function have been eagerly studied [7].

A particular focus in recent studies has been the complete regeneration of hyaline cartilage covering the subchondral bone in OA. Several treatments for damaged bone and cartilage, including mosaicplasty [10], microfracture [11], and autologous chondrocyte implantation [12], have been used in patients to relieve pain and improve joint function. However, there are several associated problems, such as limitations of available donor sites based on the required size and shape of the osteochondral autograft [9], dedifferentiation of chondrocytes during passaging in culture [13]. To solve these problems, mesenchymal stem cells (MSCs) have recently received increasing attention as promising options for osteochondral regeneration [14,15]. In numerous previous studies, chondrogenic differentiation ability was evaluated in vitro, especially using MSCs derived from bone marrow (BM), adipose tissue (AT), and other sources [15,16,17]. Among these stem cells, AT-derived MSCs (AT-MSCs) can be isolated most abundantly, and their cellular proliferation rate may be higher in mature animals [15,18]. Although it has been reported that AT-MSCs rarely differentiate into chondrocytes [19,20], AT-MSCs have been specifically shown to differentiate into cartilage in vitro [16]. Additionally, liposuction, a surgical method used to aspirate AT, is commonly utilized in the cosmetic field and is a globally accepted method for obtaining AT [21,22]. Therefore, the advantages of using AT-MSCs for cartilage regeneration are not only that 1) the stem cells can differentiate into chondrocytes but also that 2) they can be isolated more abundantly than other mesenchymal stem cells. Furthermore, 3) the cell proliferation rate is very high, and 4) they can be collected by liposuction accepted worldwide. However, conventional methods for transplanting cell suspensions have been unsuccessful in reconstructing osteochondral defects in large animals because MSCs administered to the defect do not settle and survive [20]. Therefore, it is considered indispensable to develop methods for providing MSCs in a three-dimensional (3D) format to ensure that cells are placed in the defect.

In recent years, advances in tissue engineering of functional articular cartilage have been facilitated by several methods using synthetic or biological scaffolds to achieve sufficient thickness and mechanical function, along with supporting cell attachment, migration, proliferation, and differentiation [23,24]. Studies involving surgical procedures using a combination of artificial bone and autologous chondrocytes seeded into a collagen scaffold have also demonstrated favorable bone and cartilage restoration compared to outcomes using chondrocyte suspensions [25,26]. Previous studies indicated that scaffolds composed of materials, such as collagen and hyaluronic acid, could be useful for promoting cell adhesion, proliferation, and chondrogenic differentiation [27,28] and might also facilitate stem cell seeding for implantation into osteochondral defects [29,30]. Bone regeneration using AT-MSCs seeded into hydroxyapatite has also been investigated [31]. However, the bone itself is notably self-restorative, while articular cartilage is less so [3,32]. While scaffold or scaffold-free systems have recently been investigated for the purpose of cartilage regeneration [23,24,25,26,27,28,29,30,33,34,35], it has been difficult to create sufficient thickness to fill in the osteochondral defects with or without using a scaffold. For example, a minimum thickness of 5 mm is necessary to be filled in a full-thickness articular cartilage defect of the knee. With or without a scaffold, it is even more challenging with the current methods to regenerate both articular cartilage and subchondral bone in the context of an osteochondral defect.

Alternatively, adherent cells grown in suspension can form aggregates (spheroids) in nature, avoiding death through cell-to-cell attachment [36]. The majority of the approaches using these spheroids incorporate a specific cylindrical mold to produce constructs of desired shapes [37,38]. Certain cell types, such as AT-MSCs, possess the capacity to synthesize and release components of the extracellular matrix (ECM), such as collagen, in vitro. Notably, this capacity is accelerated under confluence or 3D culture conditions because the cell cycle stops, and the cells produce ECM components. As this phenomenon has been confirmed in studies using AT-MSCs, a novel method for fusing the spheroids by using a specific cylindrical “mold” was developed to fabricate scaffold-free 3D columnar constructs consisting of AT-MSCs in vitro [39,40,41]. Two studies have reported the regeneration of articular cartilage and subchondral bone using these constructs consisting of autologous AT-MSC spheroids in minipigs [39,40]. Other studies have evaluated the outcome of using autologous AT-MSCs for osteochondral regeneration, as well [42,43]. In contrast, the use of allogeneic AT-MSCs of large animals has not yet been reported, although previous studies demonstrated the safety of the immune reaction and oncogenesis risk when using allogeneic MSCs. Besides, these cells offer some advantages, including avoidance of donor site morbidity and reducing overall cost [44,45]. Despite this, data related to whether osteochondral defects could be healed histologically by implanting 3D cell constructs made of allogeneic AT-MSCs engrafted into osteochondral defects are only available in rabbit models [41]. Moreover, to create more sophisticated constructs, the use of automatic spheroid deposition using bio-3D printing with a specific needle-array, termed the “Kenzan” method, is recommended [46]. Bio-3D printing constitutes a technology to create tissues and organs with an internal structure by 3D-stacking of spheroids at appropriate positions in the array, according to the original design. The “Kenzan,” which plays the role of a temporary artificial scaffold to fix the position of the spheroids placed by bio-3D printing, is removed prior to implantation after the spheroids fuse using the increased ECM. With this technique, spheroids are uniformly dispensed in contact with one another at a regular distance. Using these advantages, a newer method for fusing the spheroids using “Kenzan” has been developed to fabricate scaffold-free 3D tubular constructs consisting of autologous AT-MSCs. These constructs have been used for the regeneration of articular cartilage and subchondral bone in minipigs [47].

In this review, recent studies, demonstrating cartilage and osteochondral regeneration following implantation of AT-MSCs, were first introduced [48,49,50,51,52,53,54,55,56]. Second, the most recent examples of assembling AT-MSCs in three dimensions with or without scaffold for cartilage regeneration were discussed [57,58,59,60]. Third, osteochondral regeneration through implantation of scaffold-free constructs, consisting of autologous swine AT-MSCs, fabricated using the “mold”, into osteochondral defects in minipigs was discussed [39,40]. Fourth, a study describing a construct comprised of allogeneic AT-MSCs engrafted into osteochondral defects in rabbit models was presented [41]. This was in addition to examples of histological examination of MSC constructs prepared by using the “mold” method [40]. Finally, a representative study was presented that evaluated the histology of AT-MSC constructs prepared by bio-3D printing using the Kenzan method and analyzed osteochondral regeneration following the autologous implantation of two swine AT-MSC constructs [47].

## 2. AT-MSCs for Cartilage Regeneration

Several studies addressing articular cartilage regeneration using AT-MSCs were reviewed here and summarized in Table 1. First, stem cell-chondrocyte interactions for cartilage regeneration modulated by a combinatorial extracellular matrix containing hydrogel were described [48]. Next, a transforming growth factor (TGF)-β3 encapsulated polylactide-co-caprolactone (PLCL) scaffold by a supercritical carbon dioxide (CO_2_)-1,1,1,3,3,3-hexafluoro-2-propanol (HFIP) co-solvent system for cartilage tissue engineering was mentioned [49]. We also introduced an approach to promote cartilage formation of AT-MSCs seeded in polylactic-co-glycolic acid (PLGA) by dynamic compression combined with the exogenous sex-determining region Y-box (SOX)-9 [50]. Subsequently, an example was described in which cartilage in an osteoarthritis model was regenerated using AT-MSC differentiated chondrocytes [51]. In addition, chondrogenesis of AT-MSCs enhanced in hyaluronic acid-modified thermoresponsive poly N-isopropyl acrylamide (HA-PNIPAAm-CL) hydrogel for cartilage regeneration was introduced [52]. We also showed an example, illustrating enrichment of CD146^+^ AT-MSCs combined with articular cartilage extracellular matrix (ACECM) scaffold that promotes cartilage regeneration [53]. Finally, we discussed cartilage regeneration in human knee osteoarthritis using autologous AT-MSCs and autologous extracellular matrix [54].

Biochemical cues provided by methacrylated ECM molecules in hydrogels, as well as the mechanical properties of hydrogels, impact chondrogenic gene expression of AT-MSCs in 3D culture [61]. Wang et al. [48] recently reported that AT-MSCs could catalyze cartilage formation by neonatal chondrocytes (NChons) when co-cultured in biomimetic hydrogels using a 3D co-culture model. AT-MSCs and NChons were co-encapsulated in 39 combinatorial hydrogel compositions with decoupled biochemical and mechanical properties to modulate stem cell-chondrocyte interactions for cartilage repair. Both chondroitin sulfate (CS) and hyaluronic acid (HA) led to robust articular cartilage matrix deposition, as shown by the intense staining of aggrecan and type II collagen. In soft hydrogels (15 kPa), CS led to the highest amount of sulfated glycosaminoglycan deposition and increased compressive moduli. Findings from this study might guide optimal scaffold design to maximize synergistic cartilage formation using mixed cell populations. However, the hydrogels supported robust new cartilage matrix deposition by mixed AT-MSC–NChons co-culture in CS and HA containing hydrogels, and the compressive modulus in cell-containing hydrogels remained substantially lower than that of native cartilage. This showed a decrease in mechanical properties of hydrogels containing mixed AT-MSCs and NChons over time, likely due to the active degradation of hydrogels by chondrocytes to make space for a new matrix. Future studies with long-term culture using leading hydrogel compositions would help validate the increase in mechanical properties of tissue-engineered cartilage over time. Using enzymatically degradable PEG hydrogels may accelerate the speed of neocartilage deposition and improve the interconnectedness of the cell deposited matrix so that the hydrogel construct will become mechanically stronger over the culture period. Therefore, future work will include the incorporation of specific ECM molecules into hydrogels in distinct spatial zones to direct cells to secrete a cartilage-specific matrix that mimics the zones of native cartilage. On the other hand, animal experiments in which the mixed AT-MSC–NChons co-cultured in CS and HA containing hydrogels are transplanted subcutaneously in mice have been performed [62]. In the future, it is expected that additional in vivo studies in the knee joint using larger experimental animals shall provide future insight.

Mechanical cues and sustained biological cues are important factors, particularly in load-bearing tissues, such as articular cartilage [63,64,65]. Carriers, including hydrogels and nanoparticles, have been investigated for the sustained release of protein drugs [66,67,68,69]. However, it is difficult to apply such carriers alone as scaffolds for cartilage regeneration because of their weak mechanical properties; they must be combined with other biomaterials that have adequate mechanical strength. Therefore, Kim et al. [49] developed a multifunctional scaffold that had similar mechanical properties to those of native cartilage and encapsulated TGF-β3 for chondrogenesis. Tissue regeneration efficiency of the TGF-β3 encapsulated PLCL scaffold was investigated using human AT-MSCs in vitro and in vivo. Based on TGF-β3 release studies, it was confirmed that TGF-β3 molecules were released by 8 weeks and remained in the PLCL matrix. Explants of TGF-β3 encapsulated scaffolds by a co-solvent system exhibited distinct improvement in the compressive E-modulus and deposition of the extracellular matrix. Long-term delivery of TGF-β3 resulted in the formation of a hyaline cartilage-specific lacunae structure and prevented hypertrophy of differentiated chondrocytes. However, there was the possibility of immune-foreign body reaction to the capsules, including TGF-β3. Therefore, it is necessary to monitor the inflammation or degradation of them in vivo for the long-term.

Dynamic compression, as a physical stimulus, is also an important factor in regulating the proliferation and differentiation of AT-MSCs [70,71]. Zhang et al. [50] demonstrated that dynamic compression combined with exogenous SOX-9 promoted chondrogenesis of AT-MSCs in a 3D porous PLGA scaffold. Although this might benefit articular cartilage regeneration, the detailed mechanism of how the combination of exogenous SOX-9 with the gradual PLGA composite scaffold affected the metabolism and distribution of AT-MSCs has been unknown. It was assumed that the increased hypoxia-inducible factor (HIF)-1α promoted the chondrogenic differentiation of AT-MSCs because HIF-1α might play a crucial role in regulating chondrogenic differentiation and proliferation of AT-MSCs. However, the lack of studies about the proper pore density to maintain HIF-1 α expression within the physiological range and in vivo animal experiments was the limitation to be further investigated.

Recently, differentiated chondrocytes (DCs) derived from AT-MSCs have been proposed as an alternative to AT-MSCs [72,73]. Latief et al. [51] investigated the regenerative potential of AT-MSCs and DCs in the repair of damaged cartilage in osteoarthritic rats. The DCs showed better survival and regeneration potential as compared with AT-MSCs in rats and, thus, might offer a better option for regeneration of osteoarthritic cartilage. However, further studies are needed to compare DCs with AT-MSCs at the molecular level to elucidate the mechanisms involved in the repair of cartilage tissue. Outcomes after implantation of DCs and AT-MSCs in vivo using large animals, such as rabbits and pigs, should be investigated.

HA, as a microenvironmental factor, can both initiate and enhance cell aggregation and the chondrogenesis of AT-MSCs and, subsequently, facilitate hyaline cartilaginous matrix synthesis [74]. Therefore, Wang et al. [52] investigated the chondrogenic inductive potential of HA-PNIPAAm-CL, which is a newly developed and modified HA hydrogel, on enhancing rabbit AT-MSC chondrogenesis in vitro. AT-MSCs/HA-PNIPAAm-CL hydrogel constructs injected in vivo showed hyaline cartilage formation in the synovial cavity of rabbits. These results suggested that the HA-PNIPAAm-CL hydrogels provided a suitable microenvironment to enhance AT-MSC chondrogenesis for articular cartilage tissue engineering applications. Furthermore, the cell tracking images of the AT-MSC/HA-PNIPAAm-CL hydrogel demonstrated the presence of injected AT-MSCs after 3 weeks of implantation, which suggested that HA-PNIPAAm-CL hydrogel might provide a biocompatible microenvironment to facilitate the survival of transplanted/injected AT-MSCs in vivo. However, further in vivo studies must determine whether this HA-PNIPAAm-CL hydrogel combined with AT-MSCs is beneficial for long-term articular cartilage regeneration in a focal defect.

CD146^+^ MSCs are the natural ancestors of MSCs, and the expression of CD146^+^ indicates greater pluripotency and self-renewal potential [75,76,77,78]. Furthermore, CD146^+^ chondroprogenitors express higher levels of an MSC-specific marker and have better chondrogenic differentiation capacity [79]. Hence, Li et al. [53] sorted a CD146^+^ subpopulation from AT-MSCs for cartilage regeneration and combined CD146^+^ AT-MSCs with the ACECM scaffold. CD146^+^ AT-MSCs exhibited good biocompatibility with the ACECM scaffold, and this combination promoted cartilage regeneration in 6 months. However, the detail of the regulation in chondrogenic differentiation of CD146^+^ and CD146^−^ cells remains to be further investigated. In addition, sufficient numbers of CD146^+^ subpopulation is needed to be obtained without requiring serial sub-culturing in vitro and should be more feasible for future clinical applications. Furthermore, the variability of CD146^+^ cells between donors should be explored to enable the selection of the proper subpopulation for therapy.

For AT-MSC injections, platelet-rich plasma (PRP) has been used as a source of growth factors and as a differentiating agent. PRP contains various growth factors, which have been shown to have positive effects on the growth and differentiation of stem cells to chondrocyte formation [80,81]. HA has been used as scaffolding and to enhance stem cell penetration of the cartilage matrix [16,82]. Pak et al. [54] demonstrated that percutaneous injections of autologous AT-MSCs and autologous ECM in the form of an adipose stromal vascular fraction, along with HA and PRP activated by calcium chloride, could regenerate cartilage-like tissue in human knee OA patients. The resulting mixture was injected weekly into the knees of three patients with OA for 3 weeks. Although this study demonstrated that their method was a safe and potentially effective minimally-invasive therapy for OA of human knees, their paper showed that a clinical study was only performed on three patients, and the study was evaluated using magnetic resonance (MR) imaging without histological analysis. Moreover, the basic data in the animal experiments that led them to conduct clinical research were not described in the paper and references. Therefore, it is necessary to carry out some additional studies for understanding the outcomes from their method scientifically.

## 3. AT-MSCs for Osteochondral Regeneration

First, we showed the fabrication and development of artificial osteochondral constructs based on the cancellous bone/hydrogel hybrid scaffold [55]. Second, we described that chemical group-dependent plasma polymerization preferentially directs AT-MSC differentiation toward osteogenic or chondrogenic lineages [56].

It is difficult to produce osteochondral composite tissue with an optimal interface between bone and cartilage tissue [83]. Therefore, the selection and compatibilities of biomaterials and cells need to be carefully investigated, screening cell attachment and differentiation on the surface chemistries [84]. Song et al. [55] fabricated an artificial osteochondral construct to treat large osteochondral defects using tissue engineering techniques. Porcine cancellous bones and chitosan/gelatin hydrogel scaffolds were used as substitutes to mimic bone and cartilage, respectively. The cancellous bone and hydrogel composite scaffold is a promising biomaterial, which shows essential physical performance and strength attributes with excellent osteochondral tissue interaction in situ. The bi-layered scaffold significantly enhanced AT-MSC proliferation compared to the cells seeded on either single scaffold. Therefore, a bi-layered composite scaffold is an appropriate candidate for the fabrication of osteochondral tissue. However, the effect of transplanting this composite scaffold into an osteochondral defect must be carefully verified in experiments using animals. Although artificial osteochondral constructs have shown promising results in terms of biochemical characteristics and morphology, its potential application in humans still requires comprehensive studies.

Nanocomposite scaffold, which encompasses polyhedral oligomeric silsesquioxane (POSS) nanoparticles within a polyurethane backbone, can support the adhesion and growth of AT-MSCs in vitro [85]. POSS with NH_2_ and COOH functionalization can be modified using plasma polymerization [86]. Moreover, allylamine modification may increase the osteogenic differentiation of AT-MSCs [87]. Therefore, Griffin et al. [56] tested the hypothesis that different modifications of chemical groups on the surface of a nanocomposite polymer could increase the adhesion of human AT-MSCs and selectively enhance their osteogenic and chondrogenic differentiation. They showed that COOH modification significantly promoted initial cell adhesion and proliferation over 14 d compared to NH_2_ surfaces. In addition, chondrogenic differentiation was enhanced, as indicated by the up-regulation of aggrecan and collagen II transcripts. In contrast, NH_2_ group functionalized scaffolds promoted osteogenic differentiation with significantly enhanced expression of collagen I, alkaline phosphatase, and osteocalcin, at both the gene and protein levels. Finally, chorioallantoic membrane grafting demonstrated that both NH_2_ and COOH functionalized scaffolds seeded with AT-MSCs were biocompatible and supported vessel ingrowth apparently to a degree greater than unmodified scaffolds. These studies showed the ability to direct AT-MSC chondrogenic and osteogenic differentiation by deposition of different chemical groups through plasma surface polymerization. Future work will be aimed at understanding how NH_2_ and COOH affect AT-MSC chondrogenesis and osteogenesis pathway. Moreover, the repair of critical size defects in long-term in vivo studies will also need to be performed to fully understand the ability of modified scaffolds to maintain the differentiated phenotype of the AT-MSCs. Besides, using plasma polymerization has the potential to functionalize other biomaterial surfaces, including metals and ceramics. However, a detailed investigation to determine the stability of chemical groups on different surfaces and optimize the plasma modification process for each biomaterial is required.

## 4. Spheroid Formation for Cartilage Regeneration Using AT-MSCs

The use of spheroid formation for cartilage and osteochondral regeneration using AT-MSCs has been reviewed in this section and summarized in Table 2. First, we introduced the preparation and characterization of directed, one-day-self-assembled millimeter-sized spheroids of AT-MSCs [57]. Second, enhanced cartilage formation via 3D cell engineering of AT-MSCs was introduced [58]. Third, microwell-mediated microcartilage-like tissue formation of AT-MSCs was presented [59]. Finally, the substrate-dependent regeneration capacity of AT-MSC spheroids derived on various biomaterial surfaces was described [60].

Spheroid preparation technology has enabled preparation of size-controlled (>1 mm diameter) cell spheroids on a large scale by merely seeding large numbers of cells on polyion complexes-coated culture dishes [88]. Three-dimensional cell spheroids prepared using no artificial scaffold materials are desirable for cell-based transplants. Iwai et al. [57] prepared one-day-self-assembled millimeter-sized spheroids of AT-MSCs by controlling the spheroid size (diameter range: 0.4–2.5 mm). Most spheroid-derived AT-MSCs were viable and produced adhesion molecules and growth factors, which play an important role in tissue regeneration. Spheroid-derived AT-MSCs could infiltrate and recellularize collagenous tissue membranes in vitro. Therefore, it was concluded that the AT-MSC spheroids developed in this study could be directly used for cell-based tissue regeneration therapy. Furthermore, the rapid scale-up process and non-cytotoxic generation of spheroids would also support other applications, such as their use as screening models for drug discovery [57]. However, the authors did not discuss what kind of organ/tissue and how the spheroids could be applied as regenerative medicine tools at all. Furthermore, what kind of drug and disease the spheroids are applied as a drug screening model should be investigated and discussed.

Pellet culture is inappropriate for large-scale culture to obtain an adequate number of cells for clinical applications; pellet culture produces only one pellet in each culture tube [89]. In contrast, cultivation using 3D bioreactors (e.g., spinner flasks) can produce a large number of cell spheroids or pellets and facilitate culture on a large-scale [90]. Yoon et al. [58] developed an effective method for large-scale in vitro chondrogenic differentiation, which is the procedure required for clinical applications, and subsequent in vivo cartilage formation of human AT-MSCs. In vitro chondrogenic differentiation of AT-MSCs was enhanced by spheroid culture compared with monolayer culture, and the enhanced chondrogenesis was probably attributable to hypoxia-related cascades and enhanced cell–cell interactions in AT-MSC spheroids. When AT-MSCs were loaded into fibrin gel and transplanted into the subcutaneous space of athymic mice for 4 weeks, in vivo cartilage formation was enhanced compared with that of monolayer cultured AT-MSCs. These results indicated that spheroid culture might be an effective method for large-scale in vitro chondrogenic differentiation of AT-MSCs and subsequent in vivo cartilage formation. However, this method is not suitable for constructing a three-dimensional tissue body because the size of the spheroid is non-uniform and small. Furthermore, the outcomes after spheroids implantation into cartilage defects using experimental animals should be further addressed.

Recently, polyethylene glycol (PEG) hydrogel microwells have been developed to manipulate 3D cell aggregates, and stem cell differentiation can be modulated by controlling the size of the cell aggregate [91]. This provides the sizes and shapes of homogeneous 3D cell aggregate in a controlled manner. Kim et al. [59] developed scaffold-free 3D micro-cartilage-like tissue via microwell-mediated cell spheroid formation and 3D dynamic chondrogenic culture in a bioreactor. Homogenous micro-cell spheroids were generated by the self-condensation of AT-MSCs in microfabricated PEG hydrogel microwells. Subsequently, chondrogenic differentiation of the micro AT-MSC spheroids was induced in the presence of TGF-β3 under dynamic 3D culture conditions using a high aspect ratio vessel bioreactor. The 3D dynamic chondrogenic culture of AT-MSCs in the bioreactor facilitated the chondrogenic mRNA expression of proteins, such as sox-9, runt-related transcription factor 2, osterix, type II collagen, and aggrecan, and the good deposition of glycosaminoglycan and type II collagen, which finally generated micro-cartilage-like tissue [59]. Therefore, the hydrogel microwell arrays could be useful for efficiently deriving initial cell condensation-mediated chondrogenic differentiation and for developing 3D cell-based micro-cartilage-like tissue with stem cells in a controlled manner. However, there is not such a small defect as large as the microtissue clinically, so it is necessary to prepare a large cartilage tissue fusing them. Nevertheless, there can be trouble in the fusion of the micro-cartilage-like tissues because the tissue may be wrapped with type I collagen, such as cartilage particles.

Different in vitro properties have been demonstrated between self-assembled spheroids and the spheroids made from the hanging drop or non-adherent Petri dish [92], but it remains unclear if the self-assembled MSC spheroids are superior to other MSC spheroids in vivo. Therefore, Huang et al. [60] cultured AT-MSCs on a non-adherent Petri dish with polyvinyl alcohol, chitosan (ChS), or chitosan-hyaluronan (ChS-HA) to form 3D spheroids. These results demonstrated that AT-MSC spheroids derived on the ChS or ChS-HA surface had greater expression of N-cadherin and better migration ability. Moreover, animal studies also revealed significantly better cartilage repair in defects loaded with ChS-HA-derived spheroids. This suggested that AT-MSC spheroids derived on different surfaces might have distinct in vitro and in vivo properties, which appeared to be associated with the surface-bound calcium and the calcium-dependent N-cadherin and C-X-C chemokine receptor type 4 signaling. However, the animal experiment presented here only serves as a pilot study to support the different chondrogenic capacities of various biomaterial-derived spheroids. One month is too short for complete cartilage repair, and also the possibility of hypertrophic differentiation at a later stage could happen. Furthermore, outcomes after implantation of the spheroids using large animals, such as pigs and dogs, require further investigation.

## 5. Osteochondral Regeneration Using Scaffold-Free 3D Constructs Produced by the “Mold” Method

Osteochondral regeneration through the implantation of scaffold-free constructs, consisting of autologous swine AT-MSCs, fabricated using the “mold”, into osteochondral defects in minipigs was reviewed here [39,40]. More specifically, a study of a construct comprised of allogeneic AT-MSCs engrafted into osteochondral defects in rabbit models was presented [41], and examples of histological examination for MSC constructs prepared by using the “mold” method were presented [40]. Details of the methods to produce scaffold-free columnar construct and associated references are summarized in Table 3.

### 5.1. Scaffold-Free 3D Constructs Produced Using “Mold”

**Figure 1:**
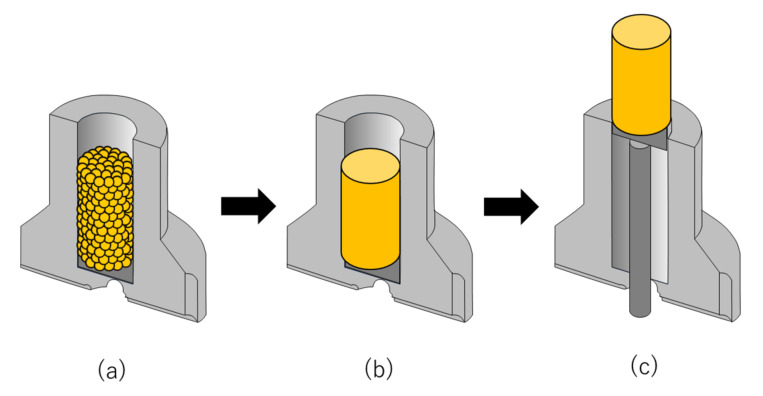
The fabrication of scaffold-free adipose tissue-derived mesenchymal stem cell (AT-MSC) construct using the “mold”. (**a**) Spheroids are piled up into a “mold” and employed to fabricate scaffold-free columnar cell constructs. (**b**) Spheroids are cultured and matured in the “mold” to fuse. (**c**) The scaffold-free cell construct is retrieved from the “mold” and used for implantation and analysis. In this figure, half of the mold is illustrated for the convenience of explanation.

Skeletally mature micromini-pigs (MMPigs) and minipigs were used in studies of osteochondral regeneration following implantation of constructs of autologous swine AT-MSC spheroids fabricated using the “mold”, as described in Table 3 [39,40]. This “mold” is made of Teflon, and the price is cheaper than 100 dollars. Liposection was performed to obtain cervical subcutaneous ATs of MMPigs with a surgical knife and a scissors [39], and Liposuction was to gain gluteal subcutaneous ATs of minipigs with a cannula and a syringe [40]. These AT samples were digested with a proteolytic enzyme, and the collected cell suspensions were cultured until construct creation. At least 4 × 10^7^ AT-MSCs were used to produce each autologous construct for both MMPigs and minipigs (Table 3). The cells were inoculated into eight low attachment 96-well plates with 5 × 10^4^ cells/well. Following incubation for 48 h, the cells formed spheroids with a diameter of approximately 700 or 500 µm in the bottom of the wells. About 760 spheroids were placed into a cylindrical “mold” (4 and 5 mm in diameter for MMPigs and minipigs, respectively) (Figure 1a; Table 3) [39,40]. The spheroids were then incubated in the “molds” in a culture medium until implantation (Figure 1b; Table 3) [39,40]. When the “mold” was carefully removed, a columnar construct for MMPigs was revealed of 4 mm in diameter and 6 mm in height (Figure 1c; Table 3) [39]. In comparison, for minipigs, the construct was 5 mm in diameter and 5 mm in height (Figure 1c; Table 3) [40]. The two types of constructs were then used for subsequent autologous implantation [39,40].

On the other hand, skeletally mature rabbits were used in a study of osteochondral regeneration following implantation of constructs of allogenic rabbit AT-MSC spheroids fabricated using the “mold”, as shown in Table 3 [41]. The constructs were made in the same manner as the swine AT-MSC constructs and used for allogenic implantation [41].

Microscopic observations of the columnar constructs revealed that the spheroids agglutinated partially with each other within the construct [39,40,41]. Numerous cell nuclei were confirmed both inside and around the spheroids without fragmentation or chromatin condensation, suggesting that the cells in the constructs were viable and did not undergo apoptosis [40]. However, it was necessary to carefully grip the constructs using surgery tweezers because ECM, such as type I collagen, was present in only small amounts [39,40,41]. The constructs showed low expression of proteoglycan, which suggested that the cells in the constructs had not differentiated into chondrocytes before implantation [40]. Based on these results, it was confirmed that AT-MSCs secreted type I collagen to form a self-generated scaffold, which migrated to fill in the interval spaces between each spheroid and then formed a columnar construct before implantation [39,40,41].

### 5.2. Swine Osteochondral Regeneration with Autologous AT-MSCs

**Figure 2:**
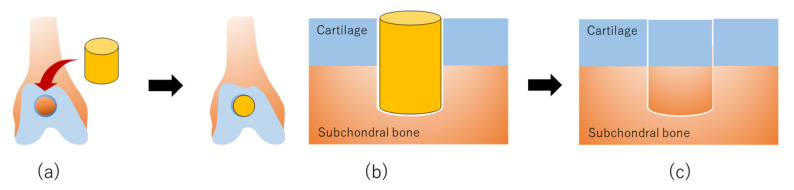
The production of scaffold-free AT-MSC constructs using the “mold” for osteochondral regeneration in vivo: (**a**) Scaffold-free cell construct and osteochondral defects are prepared for implantation. (**b**) A scaffold-free cell construct is implanted into an osteochondral defect. (**c**) AT-MSCs in the construct are differentiated to chondrocytes and osteocytes, followed by regeneration of articular cartilage in the surface layer and formation of subchondral bone in the deep layer at the implanted site.

Using a surgical trephine with an outer diameter of 4 mm, articular cartilage and subchondral bone were drilled to a depth of 6 mm at the center of the groove in MMPigs [39]. After removing a column of cartilage and bone, a cylindrical osteochondral defect was created in each groove (Figure 2a) [39]. The columnar construct was autografted into the osteochondral defect in the right hind limb (Figure 2b), whereas no graft was implanted into the defect in the left limb as control defects [39]. Alternatively, the surgery was performed under general anesthesia to create a cylindrical osteochondral defect (5 mm in depth) at the center of the groove using a bone chisel with an outer diameter of 5.2 mm in minipigs (Figure 2a) [40]. The columnar construct was carefully autografted into the osteochondral defect in one of the two defects as an implanted defect (Figure 2b), and nothing was implanted into the second defect as a control [40]. Pigs were scanned by computed tomography (CT) every 3 months following implantation and then euthanized at 24 or 48 weeks to confirm the osteochondral regeneration process by macroscopic and histological evaluation [39].

CT images of MMPigs revealed a reduction in the subchondral radiolucent area of the implanted site that became more dramatic at 2 or 3 months post-surgery compared with that at the control site [39]. A radiopaque area emerged from the boundary between the bone and the implant, and the area increased more steadily upward and inward for the implanted defect as time passed until 6 months post-surgery, compared with the control site. Thereafter, the radiopaque area of the implant gradually progressed and then filled the entire osteochondral defect at 12 months post-surgery. In contrast, in the control site, a radiopaque area emerged in the shallow layer, but the subchondral bone formation was not completed to any degree in the deep layer. Macroscopic examination at 6 months post-implantation revealed that the surface of the implanted defect was covered with abundant cartilaginous white tissues, whereas cartilaginous tissue was scarce, and the surface was depressed in the control site.

Histopathological sections showed that thickened fibrocartilage had developed over the subchondral bone regenerating in the implanted site [39,40]. The surface of the cartilage was smooth, and the boundary with the surrounding normal cartilage was obscure in the implanted site. In comparison, the surface was collapsed and irregular in the control site. At 12 months post-implantation, the surface was uniformly covered with abundant cartilaginous white tissues, and the boundary of the surrounding normal cartilage was unclear in the implanted site upon macroscopic examination [39,40]. Histological examination indicated that the surface of the cartilage was smooth, and the boundary with the surrounding normal cartilage was obscure, although small areas of endochondral ossification persisted at the center of the implanted site (Figure 2c) [39,40]. Additionally, the subchondral bone was symmetrically reconstructed in the implanted defect and was covered by a mixed matrix of hyaline cartilage and fibrocartilage (Figure 2c) [39,40]. In the control site, although fibrocartilage had immediately covered the defect, the subchondral ossification was poor [39,40].

### 5.3. Rabbit Osteochondral Regeneration with Allogenic AT-MSCs

A study of an allogeneic AT-MSC construct engrafted into osteochondral defects was carried out using skeletally mature female Japanese white rabbits as the cell donor and defect recipients (Table 3) [41]. AT-MSCs were extracted from the AT of the interscapular fat pad of the donor female [41]. The harvested AT was washed, cut into small pieces, and digested in 0.12% type I collagenase, and the resulting solution was filtered and centrifuged [41]. The pellet was resuspended in culture medium containing DMEM with 10% FBS and then plated onto culture dishes and cultured for one week [41]. To generate AT-MSC spheroids, the cells were seeded into low attachment 96-well plates at 5 × 10^4^ cells/well (Table 3) [41]. Two days following incubation, the cells aggregated into a spheroid formation approximately 700 mm in diameter (Table 3) [41]. About 800 spheroids were added into a “mold” of 4.6 mm in diameter in culture medium (Figure 1a; Table 3) [41]. The loaded spheroids adhered to one another and formed an AT-MSC construct after further culturing for several days (Figure 1b) [41]. The implant surgery was performed using aseptic techniques, and an osteochondral lesion (4.8 mm diameter and 3 mm depth) was created at the center of the trochlear groove using a drill (Figure 2a) [41]. For the implanted group, the allogenic AT-MSC constructs were gently implanted into the defect (Figure 2b) [41]. In the control group, the osteochondral defect was left empty [41]. Rabbits were euthanized 4, 8, and 12 weeks following implantation for macroscopic and histological evaluation of healed tissues [41].

The microscopic findings of the osteochondral defects revealed maturation of articular cartilage at four weeks, which increased gradually over time in the experimental group (Figure 2c) [41]. In contrast, the surface of the created defect was covered with fibrous tissue, and healing tissue with large fissures was observed at 4 weeks post-operation in the control group [41]. Therefore, the experimental group showed greater evidence of integration through cartilage-like tissue. In addition, inflammatory cells were not detected in the implanted defects. In comparison, fissures partially filled with fibrous tissue were observed in the control group.

## 6. Osteochondral Regeneration Using Scaffold-Free 3D Constructs Produced by the “Kenzan” Method

AT-MSC constructs prepared using bio-3D printing and “Kenzan” were analyzed for osteochondral regeneration following autologous implantation of two swine AT-MSC constructs as reviewed below [47]. Details of the methods to produce scaffold-free tubular construct and associated references are summarized in Table 3.

### 6.1. Scaffold-Free 3D Constructs Produced Using “Kenzan”

**Figure 3:**
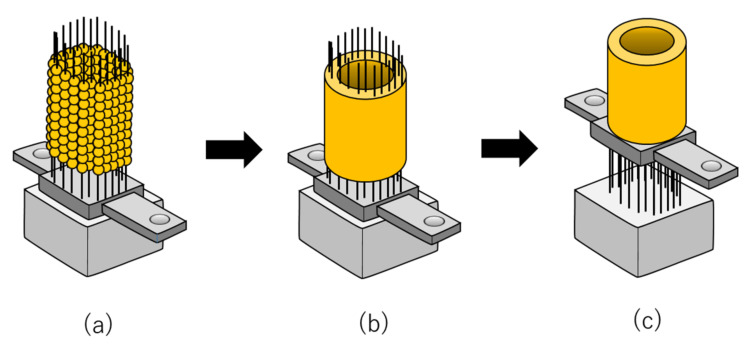
The fabrication of scaffold-free AT-MSC constructs using the “Kenzan”. (**a**) Spheroids are skewered onto the “Kenzan” automatically using a bio-3D printer and employed to fabricate scaffold-free tubular cell constructs. (**b**) Spheroids are cultured on the microneedles of the “Kenzan” to fuse with each other. (**c**) The scaffold-free cell construct is retrieved from the “Kenzan” and additionally cultured on tubular support for further maturation.

Autologous AT was aseptically excised from skeletally mature male minipigs under general anesthesia (Table 3) [47]. The AT was immediately minced and digested in collagenase, and the collected cells were resuspended in a special culture medium in which a xeno-free and serum-free MSC culture medium and a reduced serum (2%) MSC culture medium were mixed (Table 3) [47]. The cells were seeded into a culture flask and cultured until construct creation, with at least 3.0 × 10^7^ autologous AT-MSCs being used to fabricate each construct. For this purpose, the AT-MSCs were resuspended in the special culture medium, and 1.0 × 10^4^ cells/well were dispensed into low attachment 96-well plates. Following incubation for 24 h, the cells gathered in the center and formed spheroids by cell adhesion with a diameter of approximately 550 mm. After the AT-MSC spheroids were prepared, a Bio-3D printer (Regenova^®^; Cyfuse Biomedical K.K., Tokyo, Japan) was used to assemble the AT-MSCs into scaffold-free tubular tissue constructs according to a 3D model predesigned on a computer system using a bio-3D designer (B3D; Cy-fuse Biomedical K.K., Tokyo, Japan) [47]. The bio-3D printer automatically skewered the AT-MSCs spheroids onto two types of “Kenzan”, comprising needle grids of 9 × 9 (small) and 13 × 13 (large) with a needle diameter of 0.17 mm and an interneedle interval of 0.4 mm (Table 3) [47]. The usable size of the small needle array was 3.4 mm^2^ × 10 mm high, and that of the large needle array was 5.0 mm^2^ × 10 mm high (Table 3) [47]. The needle of “Kenzan” is made of tungsten, and the price is about 1000 dollars. In this system, the MSC spheroids were picked up separately from the 96-well plate by a robotically controlled fine suction 26-gauge nozzle. Sequentially, the nozzle was inserted into the needle array to skewer the spheroid onto the “Kenzan” (Figure 3a). To create two constructs according to the predesigned tubular configuration, a total of 3000 spheroids were prepared. After skewering the spheroids onto the “Kenzan”, they were placed into the perfusion chamber and cultured in the special culture medium for 1 week to fuse the spheroids (Figure 3b). The flow rate of the medium was 4–4.5 mL/min. After the spheroids were fused, constructs were extracted from the “Kenzan”, revealing a small tubular construct of 3.5 mm diameter, 4 mm height, and 1.5 mm thickness and a large construct of 5 mm, 4 mm, and 1.5 mm, respectively (Figure 3c; Table 3).

Histology of the small tubular constructs showed that the spheroids were fused within the construct, and sufficient type I collagen was produced in the constructs and almost filled in the spaces between the spheroids [47]. The constructs exhibited moderate hardness so that they could be gripped easily with surgery tweezers. The constructs also exhibited low expression of proteoglycan, whereas the majority of the cells in the construct were negative for TdT-mediated dUTP nick end labeling (TUNEL) staining with very few positive cells [47]. Based on these results, it was confirmed that AT-MSCs secreted abundant type I collagen to form a self-generated scaffold that formed a rigid columnar construct.

### 6.2. Swine Osteochondral Regeneration with Autologous AT-MSCs

**Figure 4:**
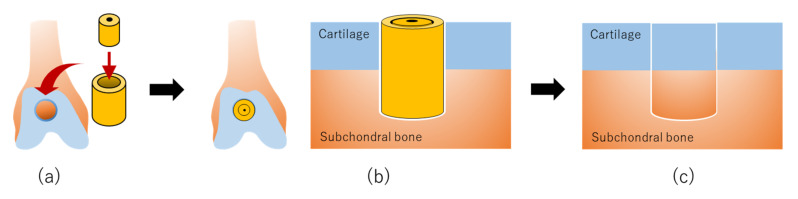
The production of scaffold-free AT-MSC constructs using “Kenzan” for osteochondral regeneration in vivo: (**a**) Scaffold-free cell construct is prepared for implantation. (**b**) A scaffold-free cell construct is implanted into an osteochondral defect. (**c**) Mesenchymal stem cells (MSCs) in the construct are differentiated to chondrocytes and osteocytes, followed by regeneration of articular cartilage in the surface layer and formation of subchondral bone in the deep layer at the implanted site.

The implant surgery was performed in pigs under general anesthesia, and articular cartilage and subchondral bone were holed to a depth of 4 mm at the center of the groove in both hind limbs using a bone chisel with an outer diameter of 5.2 mm (Figure 4a) [47]. A large tubular construct was inserted into the defect site in the right hind limb, with a small construct inserted inside the large construct to complete the graft (Figure 4b). The left hind limb was left untreated as a control. Pigs were scanned by CT and MR imaging every three months following implantation and then euthanized at 24 weeks to confirm the osteochondral regeneration process by macroscopic and histological evaluation.

A radiopaque area was found at the boundary between the bone and the graft, which increased steadily upward and inward in the defect sites implanted with constructs as compared with the control sites [47]. In both implanted and control defect sites, the area was reduced at three and six months post-surgery as compared with the area immediately after surgery. Besides, the area at three months was lower in the implanted defects than that in the control defects. MR images of the implanted defect sites showed restoration of the articular cartilage, with signal patterns similar to that of the surrounding normal cartilage. Nevertheless, new bone formation under the cartilaginous tissue was incomplete. The distinction between the superficial and deep cartilage layers, as shown by the high and the low signal intensity layers, respectively, was nearly restored, and AT predominantly occupied the subchondral area in the implanted sites. Macroscopic examination revealed that the surfaces of the osteochondral defects were better restored in the implanted sites than those of the control sites. Histopathology showed smooth hyaline cartilage, and subchondral bone formation was also noted in the implanted sites (Figure 4c). In contrast, a deeply recessed surface and a lower rate of bone formation were observed in the control defects.

## 7. Conclusions

In this review, recent studies, demonstrating cartilage and osteochondral regeneration following implantation of AT-MSCs [48,49,50,51,52,53,54,55,56], were described. This included the latest examples of AT-MSCs assembling in three dimensions with or without scaffold [57,58,59,60]. Osteochondral regeneration through implantation of scaffold-free constructs of autologous swine AT-MSCs fabricated using the “mold” was also detailed [39,40]. A construct comprised of allogeneic AT-MSCs engrafted into osteochondral defects in rabbit models was presented [41]. In addition, examples of histological examination for MSC constructs prepared by using the “mold” method were described [40]. Finally, a representative study was indicated that evaluated the histology of AT-MSC constructs prepared using bio-3D printing using the Kenzan method and analyzed osteochondral regeneration following the autologous implantation of two swine AT-MSC constructs [47].

By using the scaffold-free MSC constructs produced by the “mold”, successful achievement of the simultaneous regeneration of articular cartilage and subchondral bone for up to one year has been confirmed in small and large animals [39,40]. Additionally, the scaffold-free allogeneic MSC constructs implanted into the osteochondral defects have survived, adhered to the defect, and regenerated articular cartilage and subchondral bone in vivo [41]. Although allogeneic cells have the limitation that they will be foreign to the immune system of the recipient, it has been suggested that AT-MSCs and their secretions afford the potential to induce immunologic tolerance [93] and that allogeneic MSC transplantation does not enhance the hyper-response of T cells against donor antigens [45]. Furthermore, implantation of an artificial scaffold-free autologous MSC construct fabricated using a bio-3D printer with the “Kenzan” method into an osteochondral defect can aid in the regeneration of articular cartilage and subchondral bone in a large animal [47].

Artificial materials may, however, induce xenobiotic reactions through the tissue immune response [94], and they may not support the completion of osteochondral regeneration [36,95]. The collagen scaffold remaining in the implanted sites for long periods can prevent the regeneration of hyaline cartilage besides promoting its re-placement of fibrous cartilage [36]. Although numerous studies have reported the successful use of various biomaterials for scaffold construction, no ideal biomaterial has yet been identified. Considering the potential influence of scaffolds on the surrounding microenvironment [96], various concerns remain that need to be solved, including immunogenicity [97,98], the long-term safety of scaffold degradation products [99], and risk of infection or transmission of disease [100]. Based on these concerns, artificial scaffolds may, in principle, be unfavorable for regenerating articular cartilage; therefore, further investigation into scaffold-free cell constructs to promote cartilage regeneration is necessary.

Nevertheless, the studies described here suggest that it will be possible to produce cell constructs exhibiting optimal functions as implants for osteochondral reconstruction by optimizing the cell types and medium for construct fabrication. Moreover, it is also expected that studies using larger osteochondral defect models will be performed, and the mechanism of articular cartilage regeneration and subchondral bone formation will be evaluated by assessing the regeneration process in detail over time. After inducing AT-MSCs to chondrocytes by the method summarized in Table 1 and producing AT-MSC spheroids with or without scaffold by the method summarized in Table 2, a cell construct having a shape suitable for the osteochondral defect is formed on the “Kenzan” using a bio 3D printer. Then, it is our hope that cell constructs fabricated by the “Kenzan” method will be applied clinically as a promising treatment for OA.

## Figures and Tables

**Table 1:** Cartilage and osteocartilage regeneration using AT-MSCs.

Target Tissue (Study Design) [Reference]	AT-MSCs	Number of AT-MSCs	Materials of a Scaffold	Shape of a Scaffold	Approximate Size of a Scaffold	Other Materials	Pros and Cons
Cartilage (in vitro) [48]	N/A	7.5 × 10^5^ cells	CS-MA	Gel	50 µL	NChons	P; CS-MA scaffold maximize synergistic cartilage formationC; CS-MA decrease the mechanical properties of the scaffold
Cartilage (in vitro) [49]	N/A	1.0 × 10^6^ cells	PLCL	Discoid	D: 9.0 mm T: 3.0 mm	TGF-β3,Fibrin gel	P; Multifunctional scaffold C; The possibility of immune-foreign body reaction to the capsules
Cartilage (in vitro) [50]	N/A	1.25 × 10^7^ cells	PLGA	Columnar	N.D.	Sox-9 gene transfection	P; Dynamic compression combined with SOX-9 and PLGA scaffold. C; N.D. of the detailed mechanism and the proper pore density
Cartilage (in vivo) [51]	Autologous DCs	1.0 × 10^5^ cells	N/A	N/A	N/A	N/A	P; High regenerative potential of DCs from AT-MSCs C; N.D. of the outcomes in vivo using large animals
Cartilage (in vivo) [52]	Allogenic	2.0 × 10^5^ cells	HA-PNIPAAm-CL	Gel	200 µL	N/A	P; Suitable microenvironment for chondrogenesis in vitro and in vivo C; N.D. on long-term regeneration
Cartilage (in vivo) [53]	Xenogenic CD146^+^	5.0 × 10^5^ cells	ACECM	Columnar	D: 3.5 mm H: 2.0 mm	N/A	P; Good biocompatibility with the scaffold C; Small amount of CD146^+^ AT-MSCs
Cartilage (Clinical) [54]	Autologous	N.D.	Autologous ECM	Liquid	~ 14 mL	Other cells, PRP, and HA	P; Good clinical outcomes of three OA patients C; Small number of cases and no histopathological outcomes
Osteocartilage (in vitro) [55]	N/A	1.0 × 10^7^ cells	Cancellous bone	Rectangular	L: 10.0 mm W: 8.0 mm H: 5.0 mm	N/A	P; Bi-layered scaffold for the fabrication of osteochondral tissue C; N.D. of transplanting effect using animals
		1.0 × 10^7^ cells	Hydrogel	Rectangular	L: 10.0 mm W: 8.0 mm H: 3.0 mm		
Osteocartilage (in vivo) [56]	Xenogenic	N.D.	POSS	Tabular	L: 2.0 mm W: 2.0 mm H: N.D.	NH_2_ and COOH functionalization, CAM	P; NH_2_ and COOH functionalization of scaffolds C; N.D. of chondro- and osteogenesis pathway of the chemicals

AT-MSCs, adipose tissue-derived mesenchymal stem cells; DCs, differentiated chondrocytes from AT-MSCs; CS-MA, chondroitin sulfate methacrylate; PLCL, polylactide-co-caprolactone; PLGA, polylactic-co-glycolic acid; HA-PNIPAAm-C, hyaluronic acid-modified thermoresponsive poly N-isopropyl acrylamide; ACECM, articular cartilage extracellular matrix; ECM, extracellular matrix; POSS, polyhedral oligomeric silsesquioxane; D, diameter; H, height; T, thickness; L, length; W, width; NChons, neonatal chondrocytes; TGF-β3, transforming growth factor-beta3; SOX-9, sex-determining region Y-box-9; PRP, platelet-rich plasma; HA, hyaluronan; CAM, chorioallantoic membrane; OA, osteoarthritis; N/A, not applicable; N.D., no data.

**Table 2:** Spheroid formation for cartilage regeneration using AT-MSCs.

Target Tissue [Ref.]	AT-MSCs (Implantation)	Number of AT-MSCs	Materials for a Spheroid Formation	Approximate Size of a Spheroid (Days)	Other Materials	Pros and Cons
Cartilage [57]	Rat (N/A)	0.5 × 10^5^ −2.5 × 10^6^ cells	PCCCD	0.4–2.5 mm (1 day)	N/A	P; One-day-self-assembled millimeter-size spheroids C; N.D. for regenerative medicine tools and drug screening models
Cartilage [58]	Human (N/A)	1.0 × 10^5^ cells	Silicon	N.D. (3 days)	TGF-β3	P; Large-scale spheroid for in vitro and in vivo chondrogenesis C; N.D. of cartilage repair in vivo
Cartilage [59]	Human (N/A)	1.0 × 10^6^ cells	Silicon	290 µm (5 days)	Chondrogenic medium ^1^	P; 3D micro-cartilage-like tissue and 3D dynamic chondrogenic culture C; N.D. of fabrication methods for large size of cartilage defect
Cartilage [60]	Rabbit (Allogenic)	5.0 × 10^4^ cells	ChS-HA	N.D.	N/A	P; ChS-HA-derived spheroids C; N.D. of long-term monitoring after implantation

AT-MSCs, adipose tissue-derived mesenchymal stem cells; PCCCD, polyion complexes-coated culture dishes; ChS-HA, chitosan-hyaluronan; TGF-β3, transforming growth factor-beta 3; N/A, not applicable; N.D., no data. ^1^ Chondrogenic medium comprised high-glucose Dulbecco’s modified Eagles medium, 10% (*v*/*v*) FBS, 100 units/mL penicillin, 100 μg/mL streptomycin, 100 ng/mL dexamethasone, 50 μg/mL ascorbic acid, 40 μg/mL L-proline, 50 mg/mL insulin-transfferin-selenium, and transforming growth factor-beta3.

**Table 3:** Fabrication conditions for the bio-3D constructs.

Animal [Ref.]	AT-MSCs	Cell Number of a Spheroid	Approximate Size of a Spheroid (2 Days)	Mold or Kenzan (Shape)	Construct Shape	Construct Size	Medium	Pros and Cons
MMPig [39]	Autologous	5 × 10^4^ cells	700 µm	Mold (Cylindrical)	Columnar	D; 4.0 mm H; 6.0 mm	DMEM + 10% FBS	P; Mold price is reasonable C; Construct is fragile
Minipig [40]	Autologous	5 × 10^4^ cells	500 µm	Mold (Cylindrical)	Columnar	D; 5.0 mm H; 5.0 mm	DMEM + 10% FBS	P; Mold price is reasonable C; Construct is fragile
Rabbit [41]	Allogenic	5 × 10^4^ cells	700 µm	Mold (Cylindrical)	Columnar	D; 4.6 mm H; 3.0 mm	DMEM + 10% FBS	P; Mold price is reasonable C; Construct is fragile
Minipig [47]	Autologous	1.0 × 10^4^ cells	550 µm	Kenzan (13 × 13 Circular)	Tubular	T; 1.5 mm iD; 2.0 mm H; 4.0 mm	Combined medium ^1^	P; Construct is elastic C; Prices of Kenzan and Bio-3D printer are much more expensive than “mold”
				Kenzan (9 × 9 Circular)	Tubular	T; 1.5 mm iD; 0.5 mm H; 4.0 mm		

MMPig, microminipig; AT-MSCs, adipose tissue-derived mesenchymal stem cells; D, diameter; H, height; T, thickness; iD, inner diameter; DMEM, Dulbecco’s modified Eagle’s medium; FBS, fetal bovine serum; P, pros; C, cons. ^1^ Combined culture medium: Xeno-free MSC culture medium and serum-free MSC culture medium at a ratio of 1:1.

## Abbreviations

ACECM	articular cartilage extracellular matrix
ACI	autologous chondrocyte implantation
AT	adipose tissue
AT-MSCs	Adipose tissue-derived mesenchymal stem cells
BM	bone marrow
CAM	chorioallantoic membrane
CT	computed tomography
CS	chondroitin sulfate
CS-MA	chondroitin sulfate methacrylate
ChS	chitosan
ChS-HA	chitosan-hyaluronan
DCs	differentiated chondrocytes from AT-MSCs
DMEM	Dulbecco’s modified Eagle’s medium
ECM	extracellular matrix
FBS	fetal bovine serum
HA	hyaluronic acid
HA-PNIPAAm-C	hyaluronic acid-modified thermoresponsive poly N-isopropyl acrylamide
HIF	Hypoxia-inducible factor
MMPigs	microminipigs
MR	magnetic resonance
MSCs	mesenchymal stem cells
NChons	neonatal chondrocytes
N/A	not applicable
N.D.	no data
OA	osteoarthritis
PCCCD	polyion complexes-coated culture dishes
PEG	polyethylene glycol
PLCL	poly lactide-co-caprolactone
PLGA	poly lactic-co-glycolic acid
POSS	polyhedral oligomeric silsesquioxane
PRP	platelet-rich plasma
SOX	sex-determining region Y-box
SVF	stromal vascular fraction
TGF	transforming growth factor
TUNEL	TdT-mediated dUTP nick end labeling
3D	three-dimensional
